# A Close Examination of the Relationship Between Self-Compassion and Depressive Symptoms

**DOI:** 10.1007/s12671-018-0891-6

**Published:** 2018-01-29

**Authors:** Angélica López, Robbert Sanderman, Maya J. Schroevers

**Affiliations:** 1 University Medical Center Groningen, Department of Health Psychology, University of Groningen, Hanzeplein 1, De Brug FA12, 9700 RB Groningen, The Netherlands; 2 Department of Psychology, Health and Technology, University of Twente, Enschede, The Netherlands

**Keywords:** Self-compassion, Self-coldness, Depressive symptoms, Interaction, Longitudinal

## Abstract

Self-compassion has shown to be beneficial for individuals’ wellbeing; in particular, it has been associated with lower levels of depressive symptoms. The purpose of this study was to further explore the association between self-compassion, as measured by the Self-Compassion Scale (SCS), and depressive symptoms, in a large representative sample of community adults (*n* = 734, Mean age = 55.7, SD = 15.2). We examined the association of depressive symptoms with the SCS total score, the SCS six subscales (i.e., self-kindness, common humanity, mindfulness, self-judgment, isolation, and over-identification), and the SCS positive and negative items (referred to as self-compassion and self-coldness, respectively). In addition, we explored the predictive ability of self-compassion, self-coldness, and the SCS six subscales on depressive symptoms both cross-sectionally and over a 1-year period of time. Finally, we sought to test the moderating role of self-compassion on the association between self-coldness and depressive symptoms. Results showed that the SCS negative items and subscales were more strongly related to depressive symptoms than the SCS positive items and subscales. Accordingly, self-coldness was a stronger predictor of depressive symptoms, cross-sectionally and over a 1-year timeframe, when compared with self-compassion. Particularly, the feeling of being isolated was shown to be strongly associated with depressive symptoms. We did not find substantial evidence for a moderating role of self-compassion on the association between self-coldness and depressive symptoms. Future research needs to determine the added value of assessing self-coldness and whether or not it is an essential part of self-compassion.

## Introduction

For more than a decade, the benefits of self-compassion, the act of treating oneself kindly when experiencing difficulties, have been widely studied. A large amount of evidence has shown that self-compassion is associated with less depressive symptomatology among student (e.g., Neff et al. 2008; Raes 2011), community (e.g., Körner et al. 2015; Van Dam et al. 2011), and clinical samples (e.g., Costa and Pinto-Gouveia 2011; Krieger et al. 2013). In addition, evidence suggests that self-compassion-based interventions significantly reduce depressive symptoms compared to control conditions, and that these gains are maintained at 6 months and 1 year follow-ups (Neff and Germer 2012).

Self-compassion can be regarded as the recognition of suffering and the desire to alleviate it (Jazaieri et al. 2013). The conceptualization of Neff (2003a) is the most commonly used within the research literature. According to Neff (2003a), self-compassion encompasses treating oneself with kindness and understanding when facing suffering, seeing one’s failures as part of the human condition rather than feeling isolated, and having a balanced awareness of painful thoughts and emotions. Most of the research on self-compassion has used the total score of the Self-Compassion Scale (SCS) (Neff 2003b), a 26-item self-report questionnaire that was developed based on Neff’s conceptualization. The SCS contains six subscales, three of which measure a compassionate approach to suffering (i.e., self-kindness, common humanity, and mindfulness) with the other three measuring the opposite, a harsh attitude towards oneself (i.e., self-judgment, isolation, and over-identification). Self-kindness entails treating oneself with tenderness and understanding in the face of suffering, common humanity refers to seeing one’s failures and difficulties as part of the large human condition, and mindfulness denotes maintaining a balance awareness of the painful experiences. Self-judgment reflects harsh and critical responses to perceived failure and personal shortcomings, isolation relates to a sense of loneliness in one’s own failure and suffering, and over-identification entails becoming absorbed with painful thoughts and feelings. The SCS uses positively worded items to assess self-kindness, common humanity and mindfulness, and negatively worded items to assess self-judgment, isolation and over-identification (Neff 2003b). These six subscales are usually summed to obtain a total score of self-compassion.

In the original study, Neff (2003a) identified and proposed a six-factor, higher-order structure for the SCS, with a general factor of self-compassion explaining the inter-correlations among the six subscales. Some studies have explored this higher-order structure, generating contrasting findings. Two studies, among a student (Chen et al. 2011), and a community and clinical sample (Castilho et al. 2015), were able to replicate the higher-order model; though several other studies did not find support for the proposed structure, implying that the use of a SCS total score is not justified (Costa et al. 2015; Hupfeld and Ruffieux 2011; Kotsou and Leys 2016; López et al. 2015; Petrocchi et al. 2014; de Souza and Hutz 2016; Williams et al. 2014). In light of this evidence, Neff (2016a) argued that a higher-order solution might not be the most appropriate structure for the SCS and subsequently suggested a bifactor model. In a bifactor model, each item loads on a general factor besides loading on its respective subscale. Recently, Neff et al. (2017) found an acceptable fit for the bifactor model across four different samples and argued that the SCS total score could still be used as an overall measure of self-compassion.

Other studies have found evidence of a two-factor structure for the SCS, with one factor being formed by the positive items and the other factor by the negative items (Costa et al. 2015; López et al. 2015). In addition, there is evidence suggesting the SCS positive and negative items have different patterns of correlations with other psychological constructs. The SCS negative items have shown moderate to strong positive correlations with negative affect, perceived stress, rumination, and neuroticism, while the SCS positive items have shown weak to moderate negative correlations with these constructs, and a stronger positive association to positive affect (López et al. 2015). Consequently, some researchers have started using the SCS positive and negative items separately: the SCS positive items as a measure of self-compassion and the SCS negative items as a measure of its opposite form. Authors have referred to the composite score of the SCS negative items as self-coldness (Gilbert et al. 2011; Körner et al. 2015; López et al. 2017), self-condemnation (Dundas et al. 2015), and self-criticism (Costa et al. 2015).

There is some evidence showing that the SCS positive and negative items relate differently to depressive symptoms. Across a series of cross-sectional studies among student samples, the SCS negative subscales (i.e., self-judgment, isolation, and over-identification) showed moderate to strong correlations with depressive symptoms while the SCS positive subscales (i.e., self-kindness, mindfulness, and isolation) only showed weak to moderate associations (Garcia-Campayo et al. 2014; Krieger et al. 2013; Mills et al. 2007; Neff et al. 2008; Ying 2009; Wasylkiw et al. 2012). Others have found that a sum score of the SCS negative items correlates strongly with depressive symptoms compared to a moderate correlation of a sum score of the SCS positive items (Gilbert et al. 2011). This evidence contrasts with past research that used the SCS total score. When using the SCS total score, self-compassion appeared to be strongly related to depressive symptoms. In a meta-analysis including 14 studies, a large effect size was found for the relationship between self-compassion (as measured by the SCS total score) and depressive symptoms, with higher levels of self-compassion associated with lower levels of depressive symptoms (MacBeth and Gumley 2012).

Moreover, two recent studies suggested that self-compassion (i.e., the sum score of the SCS positive items) might moderate the relationship between self-coldness (i.e., the sum score of the SCS negative items) and depressive symptoms. In a large adult sample, representative of the German general population, Körner et al. (2015) found a significant interaction effect of self-compassion and self-coldness on depression scores, with self-coldness relating more weakly to depressive symptoms among individuals high in self-compassion than among individuals low in self-compassion. Similarly, Dundas et al. (2015) found that self-compassion moderated the association of self-coldness and depressive symptoms in a sample of undergraduate students and also found that this association became less strong at higher levels of self-compassion. Based on their findings, the authors from these studies suggested a buffering role of self-compassion on the relationship between self-coldness and depressive symptoms.

The purpose of this study was to further explore the association between self-compassion, as measured by the SCS, and depressive symptoms. In order to increase the generalizability of our findings and overcome the limitations of previous studies, we used a large representative sample from the general population and two-time measurement points. Previous research has shown contrasting findings depending on the use of the SCS. Taking this into account, we explored the association of depressive symptoms with the SCS total score, the SCS six subscales (i.e., self-kindness, common humanity, mindfulness, self-judgment, isolation, and over-identification), and the SCS positive and negative items (also referred to as self-compassion and self-coldness in the present study). In this way, we intend to gain insight about the influence of the different uses of the SCS in the understanding of the relationship between self-compassion and depressive symptoms. We also explored the predictive ability of self-compassion and self-coldness and of the SCS six subscales on depressive symptoms, both cross-sectionally and over a 1-year time-period. Finally, we sought to test the proposed moderating role of self-compassion on the association between self-coldness and depressive symptoms. Based on previous research, we expected to find stronger associations between the SCS negative items/subscales and depressive symptoms than between SCS positive items/subscales and depressive symptoms. Accordingly, we expected self-coldness to be a stronger predictor of depressive symptoms, both cross-sectionally and longitudinally, when compared with self-compassion. Finally, we expected to find an interaction effect between self-compassion and self-coldness in the prediction of depressive symptoms both cross-sectionally and over-time.

## Method

### Participants

The study was conducted among 734 individuals from the Dutch general population. The sample included 407 women (55.4%) and 327 men (44.6%). Participants’ mean age was 55.7 years old (SD = 15.2), ranging from 20 to 90 years old; 49.8% of the sample was middle educated, followed by high (34.3%) and low educated (15.9%). The majority of the sample was married or with a partner (80.9%), with the rest being single, widowed, divorced, or other (19.1%).

### Procedure

A community-based sample was selected from the civil registry offices of five middle size cities in The Netherlands. The sample was selected so as to reflect the overall Dutch population in both age and gender distributions. Having obtained the names and addresses from the municipalities, people were sent a letter with brief information about the focus of the study (i.e., self-compassion, mindfulness, and quality of life) and with the invitation to participate by filling out a self-report questionnaire. They were also sent an informed consent document, the self-report questionnaire package, and a return envelope, so they could return the informed consent document and questionnaire without any cost. In the informed consent, participants were asked for permission to approach them 1 year later in case they wanted to participate in a follow-up assessment.

For the baseline assessment (Time 1; T1), a total of 7492 persons were approached, of whom 24.4% agreed to participate and returned the informed consent and self-report questionnaire. Participants that failed to complete 15% or more of the questionnaire package were excluded. A total of 1736 adults constituted the baseline sample. One year later, the self-report questionnaire was sent to 1060 participants who gave consent to participate in the follow-up assessment (Time 2; T2). Data were obtained from 734 participants who constituted the follow-up sample. This study focuses on these 734 persons. The follow-up sample (*n* = 734) did not significantly differ from the non-respondent sample (*n* = 1002) in age or gender distributions. We did find significant differences between the two groups in education (*p* < .001) and marital status (*p* < .05); individuals with more education, married or with a partner were more likely to participate in the follow-up. The follow-up sample is representative of the general Dutch population in age and gender distributions.

### Measures

#### Self-Compassion

The 24-item Dutch version of the Self-Compassion Scale (SCS; Neff 2003b; Neff and Vonk 2009) was used to measure self-compassion. Neff and Vonk (2009) translated the original SCS into Dutch, removing 2 of the 26 items from the original English version, due to difficulties in translation. The SCS is divided into six subscales: 4-item self-kindness (e.g., “I am kind to myself when I am experiencing suffering”), 4-item self-judgment (e.g., “I am intolerant and impatient towards those aspects of my personality I don’t like”), 4-item sommon humanity (e.g., “I try to see my failings as part of the human condition”), 4-item isolation (e.g., “When I think about my inadequacies it tends to make me feel more separate and cut off from the rest of the world”), 4-item mindfulness (e.g., “When I fail at something important to me I try to keep things in perspective”), and 4-item over-identification (e.g., “When I fail at something important to me, I become consumed by feelings of inadequacy”). Self-kindness, common humanity, and mindfulness contain positively formulated items while self-judgment, isolation, and over-identification contain negatively formulated items. The items can be rated on a five-point likert scale with 1 indicating *almost never* and 5 indicating *almost always*. In the present study, the sum score of the positively formulated items was used as a measure of self-compassion, and the sum score of the negatively formulated items as a measure of self-coldness (see Costa et al. 2015; Dundas et al. 2015; Körner et al. 2015; López et al. 2015). The score of these scales may range from 12 to 60 with higher scores indicating greater self-compassion and self-coldness, respectively. The SCS total score was also calculated, by summing up all 24 items, after reversing the 12 negative items. The SCS total score can range from 24 to 120 with higher scores indicating greater self-compassion. Finally, scores were calculated for each of the SCS six subscales (i.e., self-kindness, common humanity, mindfulness, self-judgment, isolation, and over-identification), ranging from 4 to 20. The internal consistency was good for self-compassion (*α* = .86), self-coldness (*α* = .90), SCS total score (*α* = .87) and isolation (*α* = .84), and acceptable for self-kindness (*α* = .70), common humanity (*α* = .68), mindfulness (*α* = .76), self-judgment (*α* = .76), and over-identification (*α* = .77).

#### Depressive Symptoms

Depressive symptoms were assessed with the Center of Epidemiologic Studies Depression Scale (CES-D; Bouma et al. 1995; Radloff 1977; Schroevers et al. 2000)*.* The CES-D is a 20-item self-report instrument designed to measure current levels of depressive symptomatology in the general population. The scale consists of 16 negatively formulated items (e.g., “I felt depressed”) and four positively formulated items (e.g., “I enjoyed life”). On a four-point likert scale, participants specify the frequency by which each symptom was experienced during the last week (0 indicating *rarely or none of the time* and 3 indicating *most of the time*). After reversing the positively formulated items, a total score can be calculated by summing the 20 items. Total scores may range from 0 to 60, with higher scores indicating more depressive symptoms. In this study, the scale showed good internal consistency at T1 (*α* = .89) and at T2 (*α* = .91).

### Data Analyses

All analyses were conducted in SPSS version 20.0 (IBM 2011). Firstly, the relationships between the variables of interest were explored using Pearson correlations. Secondly, two multiple regression analyses were conducted to examine the predictive role of self-compassion (i.e., SCS positive items) and self-coldness (i.e., SCS negative items) on depressive symptoms at T1 and T2. The analyses were controlled for demographic characteristics that showed to be related to self-compassion and/or self-coldness, and to depressive symptoms at T1/T2 (i.e., gender and education). Thirdly, two multiple regression analyses were conducted to explore the predictive value of self-kindness, common humanity, mindfulness, self-judgment, isolation, and over-identification on depressive symptoms at T1 and T2. The analyses were controlled for demographic characteristics that showed to be related to self-kindness, common humanity, mindfulness, self-judgment, isolation and/or over-identification, and to depressive symptoms at T1/T2 (i.e., gender and education). Fourthly, and in order to test for the possible moderation effect of self-compassion on the association between self-coldness and depressive symptoms at T1, a multiple regression analysis was performed in which depressive symptoms at T1 was regressed onto mean-centered self-compassion, mean-centered self-coldness, and their interaction. Lastly, a multiple regression analysis was conducted to examine the interaction of self-compassion and self-coldness on depressive symptoms over time; depressive symptoms at T2 was regressed onto mean-centered self-compassion, mean-centered self-coldness, and their interaction. Simple slope analyses were conducted to explore the significance of the moderation slopes (Aiken et al. 1991).

## Results

### Descriptive Statistics

Means, standard deviations, and inter-correlations of all study variables are presented in Table 1. Overall, participants had average levels of self-compassion (e.g., *M* = 3) and self-coldness (e.g., *M* = 2). Participants’ mean levels of depressive symptoms were much below the cutoff for depression (> 16). These scores were as expected for a sample from the general population. Self-compassion and self-coldness had a weak negative correlation. Self-compassion showed weak negative correlations with depressive symptoms at T1 and T2. Self-coldness had strong positive associations with depressive symptoms at T1 and T2. Accordingly, self-kindness, common humanity, and mindfulness demonstrated weak negative correlations with depressive symptoms at T1 and T2, while self-judgment, isolation, and over-identification had moderate to strong correlations with depressive symptoms at T1 and T2. The SCS total score had moderate to strong negative associations with depressive symptoms at T1 and T2.

**Table 1:** Means, standard deviations (SD), and inter-correlations between study variables

		Mean	Range	*SD*	1	2	3	4	5	6	7	8	9	10
1	SCS Total	81.05	24–120	12.92	–									
2	SCS Pos	36.96	12–60	7.77	.71^***^	–								
3	SCS Neg	27.91	12–60	9.18	− .81^***^	− .16^***^	–							
4	SK	11.86	4–20	2.98	.67^***^	.85^***^	− .21^***^	–						
5	CH	12.09	4–20	3.14	.51^***^	.84^***^	.00	.55^***^	–					
6	M	13.00	4–20	2.99	.66^***^	.87^***^	− .19^***^	.65^***^	.58^***^	–				
7	SJ	10.35	4–20	3.42	− .67^***^	− .12^***^	.84^***^	− .25^***^	.02	− .10^**^	–			
8	I	8.66	4–20	3.71	− .74^***^	− .16^***^	.91^***^	− .17^***^	− .04	−. 21^***^	.61^***^	–		
9	OI	8.89	4–20	3.30	− .71^***^	− .13^***^	.90^***^	− .14^***^	.01	− .21^***^	.61^***^	.77^***^	–	
10	CES-D T1	8.96	0–60	8.05	− .52^***^	− .24^***^	.53^***^	− .26^***^	− .08^*^	− .26^***^	.39^***^	.50^***^	.49^***^	–
11	CES-D T2	9.03	0–60	8.60	− .45^***^	− .21^***^	.46^***^	− .23^***^	− .08^*^	− .22^***^	.31^***^	.46^***^	.43^***^	.69^***^

*SCS Total* Self-Compassion Scale total score, *SCS Pos* Self-Compassion Scale’s positive items (self-compassion), *SCS Neg* Self-Compassion Scale’s negative items (self-coldness), *SK* self-kindness, *CH* common humanity, *M* mindfulness, *SJ* self-judgment, *I* isolation, *OI* over-identification, *CES-D* Center of Epidemiologic Studies Depression Scale, *T1* time 1, *T2* time 2

^***^p < .001; ^**^p < .01; ^*^p < .05

### Multiple Regressions

Self-compassion and self-coldness significantly predicted depressive symptoms at T1, though self-coldness (*β* = .49, *p* < .001) was a stronger predictor than self-compassion (*β* = − .15, *p* < .001). When controlling for depressive symptoms at T1, self-coldness (*β* = .13, *p* < .001) significantly predicted depressive symptoms at T2, indicating that greater levels of self-coldness predict greater levels of depressive symptoms over a 1 year time. In contrast, self-compassion (*β* = −.04, *p* = .191) did not significantly predict depressive symptoms at T2 (Table 2).

**Table 2:** Prediction of depressive symptoms at T1 and T2 by self-compassion, self-coldness, and the SCS six subscales

	Depressive symptoms T1^a^			Depressive symptoms T2^b^		
	Beta	*p*	*R* ^2^	Beta	*p*	*R* ^2^
SCS positive and negative items models			.307			.487
Self-compassion	− .15	.000		− .04	.191	
Self-coldness	.49	.000		.13	.000	
SCS six subscales models			.320			.491
Self-kindness	− .14	.002		− .07	.069	
Common humanity	.05	.181		.02	.669	
Mindfulness	− .02	.072		.02	.687	
Self-judgment	.06	.170		− .06	.083	
Isolation	.26	.000		.13	.004	
Over-identification	.21	.000		.08	.090	

Standardized beta values are reported

^a^Analyses were controlled for gender and education

^b^Analyses were controlled for depressive symptoms at T1, gender and education

With regard to the SCS six subscales, self-kindness, isolation, and over-identification predicted depressive symptoms at T1, with isolation (*β* = .26, *p* < .001) being the strongest predictor, followed by over-identification (*β* = .21, *p* < .001) and self-kindness (*β* = − .14, *p* < .01). When controlling for depressive symptoms at T1, isolation (*β* = .13, *p* < .01) was the only significant predictor of depressive symptoms at T2, indicating that greater levels of isolation predict greater levels of depressive symptoms over a 1 year time (Table 2).

### Multiple Regressions with Interactions

The interaction term of self-compassion by self-coldness significantly predicted depressive symptoms at T1 (*β* = − .09, *p* < .01, Δ*R*^2^ = .007) (Table 3), suggesting that the effect of self-coldness on depressive symptoms can vary as a function of levels of self-compassion. A simple slope analysis revealed that the association of self-coldness with depressive symptoms was significant among participants who were low in self-compassion (*β* = .54, SE = .03, *p* < .001) as well as those high in self-compassion (*β* = .39, SE = .04, *p* < .001), with a somewhat stronger relationship among those low in self-compassion (1 SD above and below the mean were used to determine high and low levels of self-compassion, respectively).

**Table 3:** Interaction between self-compassion and self-coldness to predict depressive symptoms at T1 and T2

	Depressive symptoms T1^a^				Depressive symptoms T1^b^			
	Beta	*p*	95% CI	*R* ^2^	Beta	*p*	95% CI	*R* ^2^
Interaction models				.314				.492
Self-compassion	− .17	.000	[− .24, − .11]		− .06	.042	[− .13, − .002]	
Self-coldness	.46	.000	[.35, .47]		.11	.001	[.04, .16]	
Self-compassion × self-coldness	− .09	.008	[− .01, − .002]		− .09	.003	[− .01, − .003]	

Self-compassion and self-coldness were mean-centered; standardized beta values are reported

^a^Analyses were controlled for gender and education

^b^Analyses were controlled for depressive symptoms at T1, gender and education

Similarly, the interaction term of self-compassion by self-coldness significantly predicted depressive symptoms at T2 (*β* = − .09, *p* < .01, Δ*R*^2^ = .006) (Table 3), suggesting that the effect of self-coldness on depressive symptoms over time can vary as a function of level of self-compassion. A simple slope analysis revealed that the association of self-coldness with depressive symptoms at T2 was only significant among participants who were low in self-compassion (*β* = .18, SE = .03, *p* < .001), and not in those high in self-compassion (*β* = .04, SE = .04, *p* = .394). However, considering that the amount of explained variance by these interaction terms was rather small, and that their beta values were quite low, it is probable that their statistical significance is primarily due to the large sample size and in turn, might not be meaningful.

## Discussion

This study explored the association between self-compassion, as measured by the SCS, with depressive symptoms in a large representative sample from the Dutch general population. To gain insight on the influence of the SCS’s use on the relationship between self-compassion and depressive symptoms, we presented results for the SCS total score, the SCS six subscales, and the SCS positive and negative items. We explored the predictive ability of the SCS positive and negative items (also referred to as self-compassion and self-coldness, respectively) and of the SCS six subscales on depressive symptoms both cross-sectionally and over a 1 year period of time. Finally, we sought to test the moderating role of self-compassion in the association between self-coldness and depressive symptoms.

A key finding of our study is that the relationship of self-compassion with depressive symptoms varies depending on the way the SCS is used. The SCS total score showed a strong negative correlation with depressive symptoms at time 1 and a moderate negative association with depressive symptoms at time 2. The SCS negative items showed this same pattern of associations with depressive symptoms. In contrast, the SCS positive items showed a weak negative association with depressive symptoms at time 1 and time 2. This suggests that the strong relationship between self-compassion and depressive symptoms suggested in previous studies (e.g., MacBeth and Gumley 2012), could be mainly accounted for by the SCS negative items that measure a hard and cold response to the self, that is, the exact opposite of self-compassion. This also implies that the positive experience of self-compassion, a kind and understanding response to the self, might only be weakly associated with depressive symptoms.

In addition, we found that self-coldness (i.e., SCS negative items) was a stronger predictor of depressive symptoms, both cross-sectionally and longitudinally, compared to self-compassion (i.e., SCS positive items). Self-coldness positively predicted depressive symptoms at time 1 and time 2, whereas self-compassion negatively predicted depressive symptoms at time 1 but not at time 2. When looking at the SCS six subscales, isolation was the stronger predictor of depressive symptoms at time 1, followed by over-identification and self-kindness; isolation was the only predictor of depressive symptoms at time 2. In another study among individuals from the general population that reported symptoms of anxiety and depression, isolation was also the strongest predictor of depressive symptoms, followed by over-identification and self-judgment (Van Dam et al. 2011). Similarly, in yet another community sample study, isolation was the strongest predictor of depressive symptoms, followed by over-identification, self-judgment, mindfulness, and self-kindness (Körner et al. 2015).

As proposed in previous studies, we found a significant interaction between self-compassion and self-coldness. Our results showed that cross-sectionally, self-coldness predicted depressive symptoms in individuals with either low or high self-compassion, with a somewhat stronger relationship among those low in self-compassion. In our longitudinal data, self-coldness predicted depressive symptoms only for those individuals low in self-compassion. These findings are in line with those of Dundas et al. (2015) and Körner et al. (2015). However, considering the amount of explained variance and beta values found for the interaction were quite low, it is highly probable that its significance is mainly explained by the large study sample. Körner et al. (2015) also reported a very low beta value (*β* = − .08) and amount of explained variance (*R*^2^ = .01) for the interaction; similarly, their study was conducted among a large sample from the general population (*n* = 2404).

Together, the findings from this study suggest that if measured as separate constructs, self-compassion and self-coldness are related differently to depressive symptoms. An important arising question is the function and value of assessing self-coldness. Self-coldness measures harsh judgment towards the self, feelings of isolation, and over-identification with negative aspects of oneself or personal experiences (Neff 2016b). These concepts resemble self-criticism, loneliness, and rumination (Muris and Petrocchi 2016), three well-known and studied psychological processes that have proved to be detrimental for individuals’ well-being (e.g., Cacioppo et al. 2006; Mor and Winquist 2002; Murphy et al. 2002). Self-criticism has been associated with lifetime risk for depression (Murphy et al. 2002). Two processes which seem to be linked with self-criticism are the incapability to resist self-attacks and the inability to generate feelings of warmth and reassurance for the self (Gilbert et al. 2006; Gilbert and Procter 2006; Kelly et al. 2009). Social isolation denotes loneliness that in turn has been found to be a risk factor for future depressive symptoms (Cacioppo et al. 2006), and found to diminish the capacity to self-regulate emotions (Hawkley and Cacioppo 2010). Lastly, over-identifying with painful thoughts and emotions implies a ruminative self-focused process that has been largely demonstrated to be positively related to negative affect (Mor and Winquist 2002). Ruminative responses prevent effective coping, enhancing, and prolonging depressive mood states (Mor and Winquist 2002).

Currently, there is an ongoing discussion on the validity of the SCS total score. Some researchers have argued that including the negative components of self-judgment, isolation, and over-identification in the measurement and conceptualization of self-compassion can “partially operationalize self-compassion as a mirror image of psychopathology” (Muris et al. 2016, p. 3). According to these authors, self-compassion can better be conceptualized in a way that truly reflects its protective qualities, instead of tapping into toxic mechanisms that do not fit with the true nature of self-compassion. Neff (2016b), however, argues that the SCS negative components are essential to self-compassion as they are part of interacting pairs (self-kindness vs self-judgment, common humanity vs isolation, mindfulness vs over-identification) that focus on the different ways in which individuals emotionally and cognitively respond to their suffering.

More research needs to be conducted in order to determine whether self-coldness has added value when studying self-compassion. Future studies could explore how the components of self-coldness (self-judgment, isolation, and over-identification) relate to the already known toxic mechanism of self-criticism, loneliness, and rumination, and consequently explore their combined and unique predictive value for psychological well-being. When studying self-coldness, it would be important to be cognizant of the various conceptualizations of its items. For instance, isolation has been studied in the broader literature as being socially isolated (an objective lack of contact with people [e.g., Brummett et al. 2001]), whereas Neff’s understanding refers more to feelings of loneliness—a subjective experience of being lonely even when surrounded by others. Future research would also greatly benefit from the use of other assessment tools to measure self-compassion. The Compassionate Engagement and Actions’ scales, a set of three scales that aim to measure three orientations of compassion (compassion to others, self-compassion, and receiving compassion from others) was recently developed and tested (Gilbert et al. 2017). This scale uses ten positively formulated items to assess self-compassionate engagement (e.g., “I notice, and am sensitive to my distressed feelings when they arise in me”) and actions (e.g., “I take the actions and do the things that will be helpful to me”).

The large community sample with equivalent gender distribution and broad age range, strengthen the generalization of our results. The use of two time points allowed the exploration of the effect of self-compassion and self-coldness on levels of depressive symptoms over time. These results are relevant due to the fact the majority of past research has used cross-sectional designs. Though it is important to notice that during the year in between measurement points, many events could occur in the lives of our participants that are not accounted for. One way to minimize this problem is the use of a shorter period in between the measurement points. Additionally, it is important to state that from the two measurement points it is not possible to draw causal inferences. In order to make more definite inferences regarding the temporal association between self-compassion and well-being, it would be necessary to measure these variables at multiple points over time (i.e., more than two measurements). Another limitation that needs to be considered is a loss to follow-up. Loss to follow-up was associated with lower educational level and not having a partner. To address this issue we checked if there was a difference between the respondent and non-respondent samples regarding the associations of self-compassion and self-coldness with depressive symptoms at baseline. These associations did not differ between the two groups. Therefore, this suggests that the loss to follow-up did not affect the results of our longitudinal studies to a major degree. Finally, it should be noted that our results might not be generalizable to clinical samples that often show elevated levels of psychological symptoms or negative affectivity when compared with community samples. Some evidence suggests differences between these populations in levels of self-compassion, with clinical samples having significantly lower levels of self-compassion than non-clinical samples (Krieger et al. 2013).

In conclusion, the strong relationship between self-compassion and depressive symptoms seems to be mainly accounted for by the negative items/subscales of the SCS (measuring self-judgment, isolation, and over-identification). Particularly, the feeling of being isolated was shown to be strongly related to depressive symptoms. Self-coldness was a stronger predictor of depressive symptoms than self-compassion, both cross-sectionally and over a 1-year period. We did not find substantial evidence for a moderating role of self-compassion on the association between self-coldness and depressive symptoms.
